# Dual-channel pulse-dye densitometry can enable correction of fluorescent targeted and control agent plasma input function differences for quantitative paired-agent molecular imaging: a simulation study

**DOI:** 10.1117/1.JBO.30.4.046001

**Published:** 2025-03-29

**Authors:** Cody C. Rounds, Yichen Feng, Sanjana Pannem, Jovan Brankov, Kimberly S. Samkoe, Kenneth M. Tichauer

**Affiliations:** a Illinois Institute of Technology, Biomedical Engineering, Chicago, Illinois, United States; b Dartmouth College, Geisel School of Medicine, Hanover, New Hampshire, United States; c Dartmouth College, Thayer School of Engineering, Hanover, New Hampshire, United States; d Illinois Institute of Technology, Electrical and Computer Engineering, Chicago, Illinois, United States

**Keywords:** paired-agent imaging, pulse-dye densitometry, ABY-029, IRDye 680LT, plasma input function

## Abstract

**Significance:**

Paired-agent fluorescent molecular imaging approaches involve co-administration of a control (untargeted) imaging agent with a molecularly targeted agent to account for non-specific effects and quantify binding potential (BP)—a parameter proportional to the concentration of the targeted biomolecule. Accurate BP estimation often requires correction for differences in targeted and control agent plasma input functions (PIFs).

**Aim:**

We provide a simulation-based evaluation of whether dual-channel pulse dye densitometry (PDD) can be used to measure the PIFs of co-administered targeted and control imaging agents, to enable accurate BP estimation.

**Approach:**

Monte-Carlo simulations of light propagation were carried out using the anatomy and optical properties of a finger, as well as experimentally measured PIFs of co-administered anti-epidermal growth factor receptor fluorescent affibody, ABY-029, and IRDye 680LT, a control imaging agent from past mouse experiments. The accuracy of PIF shape estimation from PDD and PIF difference correction was evaluated by assessing BP estimation accuracy in a simulated “tumor” tissue.

**Results:**

“Tumor” BP measurements using deconvolution correction with noise-free PIFs versus PDD-measured PIFs were compared. The relative error in PDD PIF deconvolution BP estimation was 2±1%. No statistical difference was found between the estimated BP via deconvolution correction with true PIFs and the estimated BP via the reconstructed PIFs using the proposed PAF-PDD methodology.

**Conclusions:**

These results highlight the potential for developing a PDD instrument that can directly measure targeted and control agent PIFs and be used to correct for any PIF differences between agents for more quantitative estimates of BP in paired-agent imaging studies.

## 1 Introduction

In recent years, paired-agent imaging (PAI) strategies have been explored owing to their ability to yield quantitative *in vivo* imaging of cell-surface receptors.^1^^–^^3^ PAI requires the administration of at least two imaging agents of similar blood and tissue kinetics where one agent is targeted to a biomolecule of interest and one is untargeted (the “control” agent). By measuring the dynamics of the control agent, non-specific accumulation of the targeted agent and variable targeted agent delivery (attributable to the variability of blood flow and vascular permeability among tissues, particularly cancerous tissues) can potentially be accounted for, allowing for more quantitative estimates of the targeted agent’s specific binding through the approximation of a quantity termed the “binding potential” (BP). BP is directly proportional to the concentration of an agent’s biomolecular target,^4^ and as such, the measurement of this quantity can give critical insight into the disease state and phenotype of a biological tissue. In the context of surgical oncology, BP mapping (compared with targeted agent distribution mapping) may enable improved discrimination between malignant and non-malignant tissue types.^5^^,^^6^

For the control (untargeted) agent to account for variable delivery and non-specific retention of the targeted imaging agent, at least four key assumptions must be made about the control agent (1) that it exhibits no binding to the biomolecule of interest and if it exhibits any “non-specific” binding, it is equivalent to that exhibited by the targeted agent; (2) that it experiences similar rates of diffusion across blood vessel walls compared with the targeted agent; (3) that the signal emitted from the control agent (e.g., fluorescence) propagates through the tissue similarly to that of the signal emitted from the targeted agent; and (4) that it has similar blood pharmacokinetics compared with the targeted agent.^3^^,^^7^ The first assumption is typically satisfied by careful selection of a non-specific moiety to label with the reporter, typically a “scrambled” version of the specific binding moiety (e.g., isotype IgG antibody, negative control affibody).^1^^,^^6^^,^^8^^–^^11^ The second assumption can be largely satisfied by ensuring that the targeted and control imaging agents have relatively similar size, charge, and lipophilicity.^12^^–^^15^ The third assumption requires a thorough understanding of the propagation of signals in biological tissue. For fluorescent agents, combining the 700-nm range with 800-nm-range emitting fluorophores for labeling the control and targeted agents, respectively, or in reverse, has been shown to enable sensitivity to similar volumes of interest in tissues with typical optical properties.^9^ In our group’s experience, the fourth assumption (that the control agent has similar blood pharmacokinetics to the targeted agent) is much more difficult to control. For one, the mere fact that the targeted agent is binding in tissues can influence the global concentration of the targeted agent in the blood. Moreover, the mechanisms of how agents are extracted from the blood are complex, potentially being mediated by multiple organs of filtration (e.g., kidney or liver), the complexity of which can be influenced by miniscule differences in the chemical properties of the targeted and control agents.^12^^,^^16^

In response, a few approaches have been developed to correct for differences in the plasma input functions (PIF) of paired targeted and control agents.^1^^,^^17^ One approach involves the selection/identification of a “reference” tissue, one devoid of the biomolecule of interest where any difference in the temporal dynamics of the control and targeted agents is assumed to reflect differences in the agents’ PIFs.^2^^,^^18^^,^^19^ With this approach, the signal over time of one agent can be represented as a convolution of the signal from the other agent and an arbitrary function, g(t).^17^ The g(t) function can be calculated by deconvolution and then used to correct for agent PIF differences in all other tissues/regions of interest (ROI) as detailed previously.^17^ Although used with some success, there are limitations to this method, including the need to assume that both agents exhibit similar likelihoods of diffusing from the tissue to the blood (“efflux”; $k_{2}$) in both the reference region and the ROI (though $k_{2}$ can vary between the ROI and the reference region), that the reference tissue is truly devoid of any of the targeted biological molecule, and that any non-specific binding effects are experienced by both agents relatively equally.^20^

If the PIFs of the targeted and control agents can be directly measured, then the deconvolution method of accounting for differences in paired agent PIFs could be carried out directly and there would be no need to find a suitable reference tissue (if one even exists).^20^ Current methods of quantifying PIFs can require vessel cannulation and serial blood draws, or direct imaging of a major artery or blood vessel, such as the carotid.^21^^–^^23^ For paired-agent fluorescence imaging approaches, the only non-invasive way to directly image signals from blood vessels is to image the retina.^24^ Alternatively, indirect measures of fluorescent imaging agent absorption in arterial blood have been developed by processing changes in bulk tissue absorption that occur at the heart rate frequency. These methods have equivalence to what is done in pulse oximetry and were originally developed by the same group who created the first pulse oximeter.^25^^,^^26^ This concept, characterized as pulse-dye densitometry (PDD), was first published in 1997 by Iijima et al.^27^ to measure the plasma concentration of the near-infrared (NIR) fluorophore indocyanine green (ICG) over time.^28^^–^^30^ The work in this paper aims to provide an initial evaluation of whether the principles of PDD can be expanded to measure the PIFs of a targeted and control agent concurrently and demonstrate that even a two-channel “finger probe” fluorescence system (i.e., a system similar to a pulse oximeter that has one channel for measuring the fluorescence of the targeted agent and another for the control agent) has the potential to estimate PIF shapes by monitoring fluorescence over time with our paired-agent-PDD approach rather than absorption. Such an approach does not enable absolute quantification of PIFs, but by normalizing relative targeted and control agent PIFs at an early time-point after injection, accurate paired agent PIF correction can be achieved as demonstrated.

## 2 Methods

To evaluate the feasibility of a finger probe-based two-fluorescence-channel system to be used to correct for targeted and control agent PIFs in PAI, prior to constructing a system, this work includes the use of MCMatlab (an open-source Monte Carlo-based photon propagation software written in MATLAB) to simulate photon propagation in a finger-like object. Arterial blood vessel expansion and contraction were simulated along with a varying concentration of a targeted agent and control agent in the arterial blood compartment matching PIFs from experimental work carried out in mice where PIFs of the targeted agent, IRDye 800CW (LI-COR Biosciences, Lincoln, NE)-labeled anti-epidermal growth factor receptor (EGFR) affibody, ABY-029, and the control agent, IRDye 680LT (LI-COR Biosciences, Lincoln, NE) were measured directly from blood samples.^31^ A PDD-like approach was then used to estimate PIF shapes, and the accuracy of the measured shapes was compared with simulated “truths” and used in a simulated tumor to evaluate the accuracy of BP estimation after deconvolution correction, all using experimentally relevant levels of noise added to all simulations.

### 2.1 Simulation of Targeted and Control Agent Fluorescence Signals as a Function of Time in the Finger

#### 2.1.1 Three-dimensional finger simulation

**Fig. 1:**
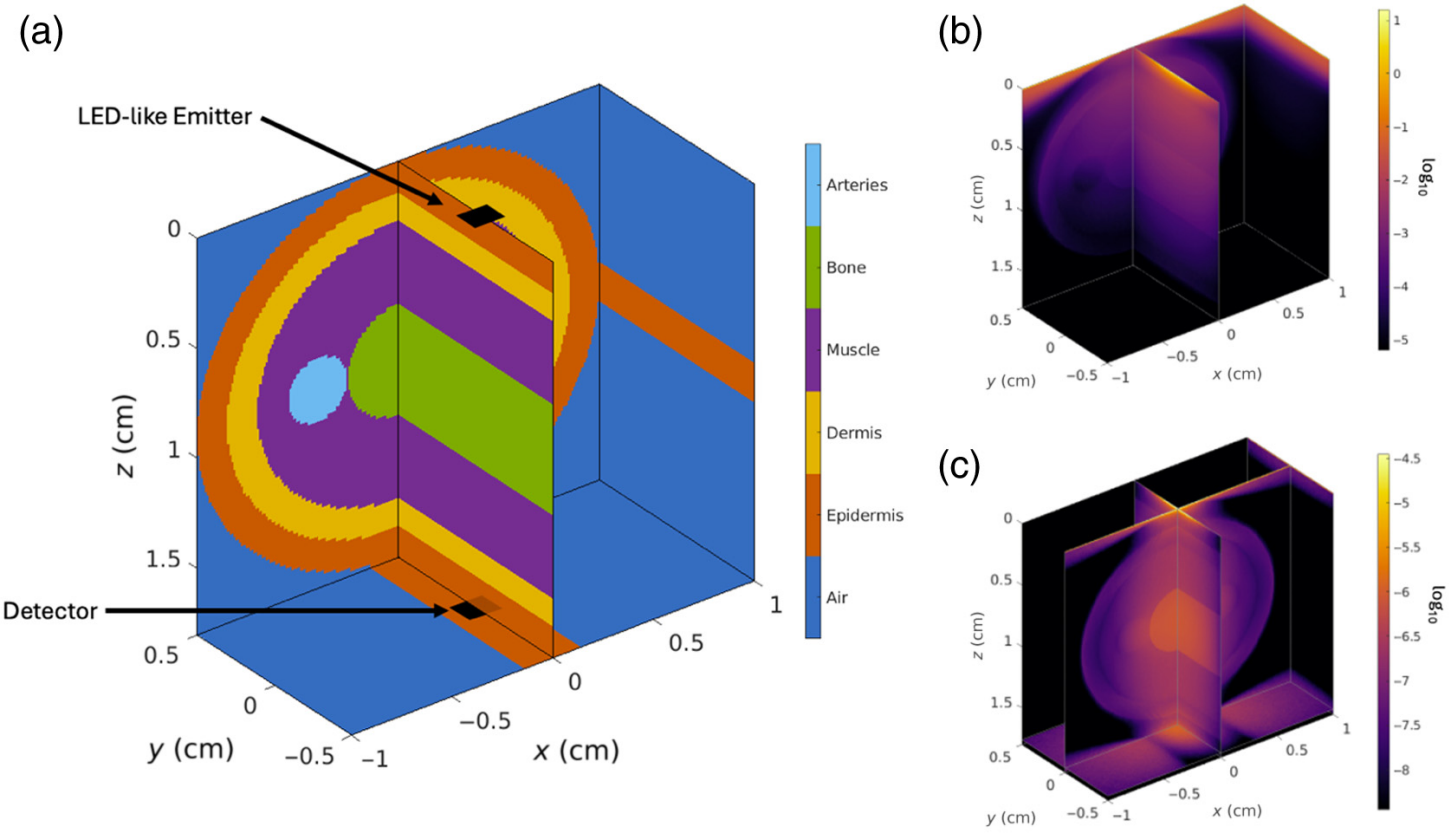
(a) Simulation geometry with the different tissue types used in the simulation, along with the depictions of the emitter and detector locations, (b) excitation simulation normalized fluence rate, and (c) complete detector sensitivity profile.

A human index finger geometry was simulated using the photon propagation Monte-Carlo modeling software, MCmatlab.^32^ MCmatlab simulates photon propagation through a cartesian finite-element mesh. Here, a realistic finger geometry was utilized as a medium through which photon propagation was simulated, assuming a light-emitting diode (LED)-like emitter at the top of the finger. A depiction of the simulated finger geometry is presented in Fig. 1(a), where the finger was defined as a 1-cm-long, 2-cm-diameter cylinder divided into five regions: epidermis (0.15 cm thick), dermis (0.15 cm thick), muscle (0.2 cm thick), bone (1 cm thick), and arterial blood (in the form of two 0.08 cm thick blood vessels). The object was divided isotropically into 200×200×200 bins in the x, y, and z axes, 10  μm in size in all dimensions. Each region was assumed to have unique and homogeneous optical properties, which are listed in Table 1.

**Table 1:** Tabulated optical properties used in the simulations for the corresponding tissue types in ${\mathrm{cm}}^{-1}$, with $\mu_{a}$ and $\mu_{s}$ depicting the absorption and scattering coefficients, respectively. The optical properties shown here are for 800 nm light. The anisotropy factor, g, was assumed to be 0.9 for all tissues.

	$\mu_{a}$ (${\mathrm{cm}}^{-1}$)	$\mu_{s}$ (${\mathrm{cm}}^{-1}$)
**Epidermis** ^33^	10	8
**Dermis** ^33^	0.2	8
**Muscle** ^34^ ^,^ ^35^	0.3	35
**Arterial blood** ^36^ [Bibr r37] ^–^ ^38^	0.3	225
**Bone** ^39^	0.2	100

Although described in the accompanying citations, it is important to clarify the sources and methodologies used to determine the optical properties used in this study. In the case of skin (epidermis and dermis), the absorption coefficients are based on a baseline measurement of neonatal skin absorption paired with the relative abundance of melanocytes and hemoglobin and their associated absorption for epidermal, dermal, and muscle layers added (with myoglobin considered the major absorber in the case of muscle). Individual absorption as well as volume fractions for these molecules was found empirically. For scattering, these parameters were calculated based on Mie theory where scattering by cylindrical collagen fibers is assumed to be dominant. Optical properties of arterial blood were calculated similarly to that of the skin, where the primary absorber was assumed to be hemoglobin and the primary scatterer was assumed to be red blood cells, which can be approximated as homogenous spheres. Optical properties used for the bone were found empirically via spectrophotometry paired with an integrating sphere and the inverse adding-doubling method. All empirical methods to determine either baseline or whole properties were done using human tissue, as is relevant for the scope of this study.

The excitation fluence profile, visible in Fig. 1(b), was calculated by simulating a $1\text{-}{\mathrm{mm}}^{2}$ LED-like top-hat emitter at the top of the cylinder and $1\times 10^{10}\;\text{photons}$ were simulated entering the top of the finger with a 90 deg half-angle exit profile. The detector fluence profile was found by taking the excitation fluence profile and rotating it 180 deg around the y-axis. The full Jacobian of the system [Fig. 1(c)] was calculated by performing a pixel-by-pixel multiplication of the excitation and detector fluence profiles, utilizing the so-called “adjoint” method.^40^

To simulate realistic fluence profiles, multiple simulations were carried out, where the optical properties of the arterial region were varied according to a simple cosine wave that was proportional to the change in arterial volume, with the periodicity corresponding to a heart rate of 60 beats per minute (BPM). The maximum diameter of the arterial component was set to 0.832 mm, and the minimum diameter was set to 0.786 mm, corresponding to a mean diameter of 0.8  mm±4%.^41^ All simulations and calculations were carried out using MATLAB vR2019a (MathWorks, Natick, MA, United States), and photon propagation simulations for different time points were parallelized across two NVIDIA GeForce RTX 2080 Ti and two NVIDIA GeForce RTX 1080 Ti (NVIDIA, Santa Clara, CA, United States) graphics processing unit (GPUs) using MATLAB’s parallel computing toolbox and the GPU acceleration feature provided in the MCmatlab software.

#### 2.1.2 Arterial concentrations of representative targeted and control fluorescent imaging agents as a function of time

Measured population average PIFs from mice after intravenous injection of a targeted agent (ABY-029) and a control agent (IRDye 680LT) were used to simulate the concentration of paired agents in the artery. These agents were chosen as an example pair of targeted and control agents that are known not to exhibit similar PIFs, where this difference is notable via the previously described bi-exponential decay functions found from group-wise blood draw studies.^31^^,^^42^ Bi-exponential representations of the PIFs for ABY-029 and IRDye 680LT are represented in Eqs. (1) and (2), respectively. The corresponding curves calculated from these equations were then used as inputs for modeling non-arterial (“tissue”) imaging agent concentrations as a function of time, as described in Eqs. (3) and (4)

$$C_{p,ABY-029}(t)=0.95\;\mu Me^{-0.23\;\min^{-1}t}+0.48\;\mu Me^{-0.0026\;\min^{-1}t},$$ (1)

$$C_{p,IRDye\;680\;LT}(t)=5.2\;\mu Me^{-0.22\;\min^{-1}t}+0.35\;\mu Me^{-0.0042\;\min^{-1}t}.$$ (2)

#### 2.1.3 Tissue concentrations of representative targeted and control fluorescent imaging agents as a function of time

**Fig. 2:**
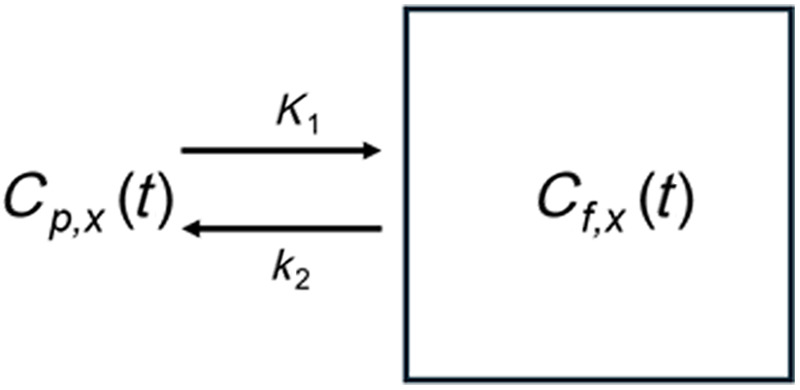
Single-compartment model used to simulate the concentration of imaging agents as a function of time in the tissue ROI. The resulting differential equation for the concentration of the tissue ($C_{f,x}$) can be seen in Eq. (3) where $C_{p,x}$ is the PIF of the corresponding fluorophore x, $K_{1}$ represents the rate of extravasation from the vessels into the tissue space, and $k_{2}$ represents the rate of flow from the tissue space back into the arterial space. The analytical solution to this model can be seen in Eq. (4).

To simulate the concentrations of the fluorophores in the non-arterial tissue regions over time, a one-compartment model was assumed. The differential equation governing this was represented by

$$\frac{dC_{f,x}}{dt}=K_{1}C_{p,x}(t)-k_{2}C_{f,x}(t),$$ (3)

where $C_{f,x}$ represents the concentration of imaging agent in the finger outside of the blood compartment, $K_{1}$ represents the rate constant governing diffusion of the imaging agent across the blood vessel wall (from blood to tissue), $C_{p,x}$ represents the corresponding PIF [see Eqs. (1) and (2)] for mathematical representations of the ABY-029 and IRDye 680LT agents, respectively), and $k_{2}$ represents the rate constant governing diffusion of the imaging agent from the tissue back to the blood. The corresponding analytical solution can then be represented as

$$C_{f,x}(t)=K_{1}C_{p,x}(t)*(u(t)e^{-k_{2}t}),$$ (4)

where “*” represents the convolution operator and u(t) represents the unit step function. Previously measured typical $K_{1}$ and $k_{2}$ were used with values of 0.1 and $0.08\;{\mathrm{cm}}^{-1}$, respectively.^2^ An illustration of this single-compartment system can be seen in Fig. 2.

#### 2.1.4 Realistic noise simulation

**Fig. 3:**
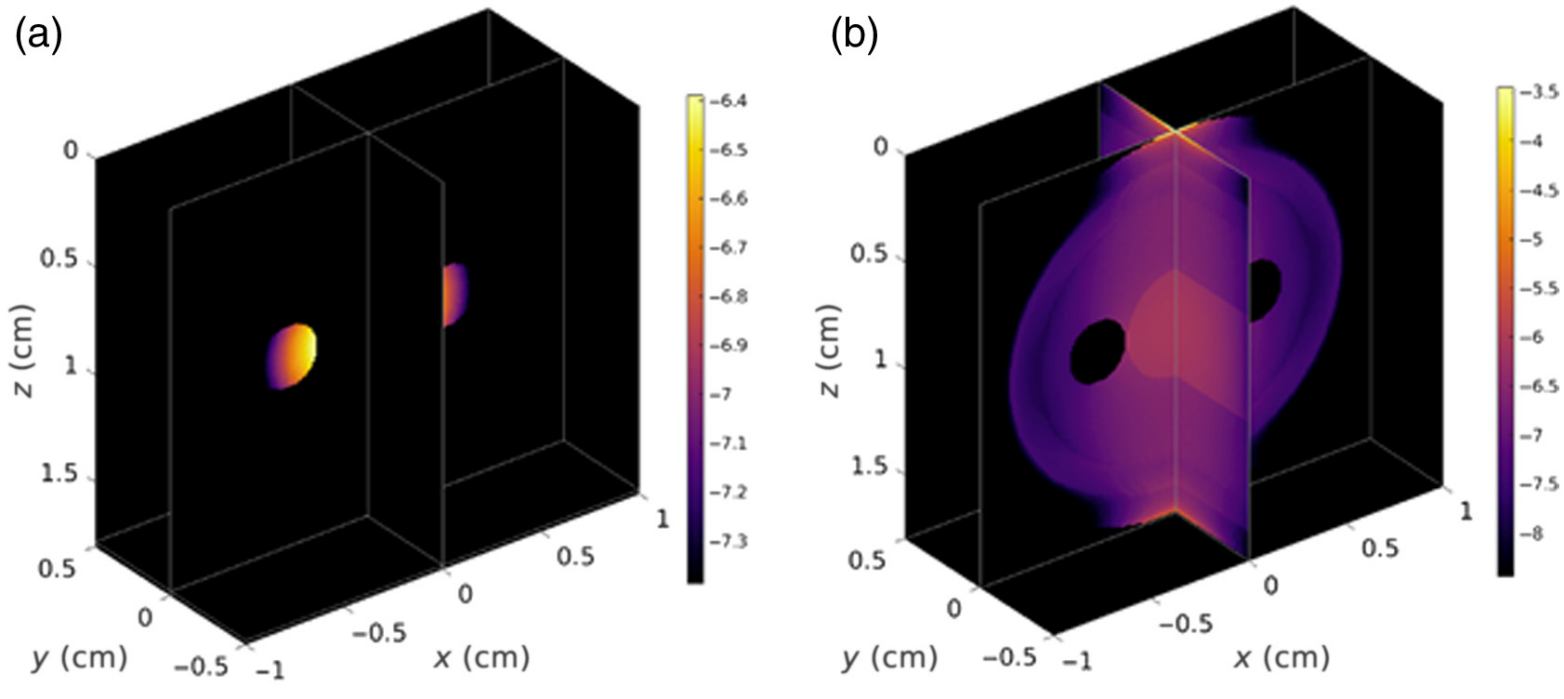
Simulated “Jacobians” of the system describing the sensitivity of the source-detector orientation to fluorescence in object locations for (a) artery ROIs and (b) tissue ROI, used to compute mean fluorescence intensities per ROI through multiplication by ROI-specific concentration curves. Fluence profiles displayed in a log-scale for visualization.

The total fluorescence signal over time was calculated by first isolating the portions of the Jacobian (probabilistic photon propagation distribution from the Monte Carlo simulations) relevant to both “tissue” and “arterial” ROIs using the simulated geometry [Fig. 3(a)] followed by scaling by the published $C_{y,x}(t)$ for each imaging agent followed by integration across all three spatial dimensions per time point as follows:

$$S_{x}(t)=\underset{i}{\sum }I_{ex}\phi_{ex}(r_{i},t)\phi_{em}(r_{i},t)\eta_{x}\varepsilon_{x}C_{y,x}(r_{i},t),$$ (5)

where $S_{x}(t)$ represents the fluorescence signal measured as a function of time, t, at the wavelength associated with imaging agent “x” (i.e., x=ABY-029 at 800 to 840 nm for example, or IRDye 680LT at 700 to 740 nm); $I_{ex}$ represents the intensity of the excitation light source; $\phi_{ex}(r_{i},t)$ and $\phi_{em}(r_{i},t)$ that represent the probabilistic distribution of the excitation light coming from the light source into the finger and the probabilistic distribution of likelihood that fluorescence emission light at a given location will enter the detector, respectively (both outputs of the Monte Carlo simulation) at each spatial location, $r_{i}$, as a function of time given the oscillating diameter of the arterial compartment at the heart rate; $\eta_{x}$ represents the quantum efficiency of imaging agent x; $\varepsilon_{x}$ respresents the molar extinction coefficient of the imaging agent at peak absorption (780 nm for ABY-029 and 680 nm for IRDye 680LT); and $C_{y,x}(r_{i},t)$ represents the concentration of the imaging agent x over time at location $r_{i}$, where everything inside the blood compartments was set to $C_{p,x}$ [Eqs. (1) and (2)], and everything outside the blood compartment was set to $C_{f,x}$ [Eq. (4)]. $C_{p,x}$ was assumed to be sinusoidal, oscillating according to a sinusoid with a frequency at the heart rate, which was set here to be 60 BPM, as described in Eq. (6), where ω corresponds to the heart rate, and α is the proportion of arterial volume change during each pulse of the heartbeat. Here, $C_{p-raw,x}(t)$ corresponds to the original PIF calculated via Eqs. (1) and (2), whereas $C_{p,x}(t)$ is the oscillatory plasma concentration used to calculate $S_{x}(t)$ in Eq. (5).

$$C_{p,x}(t)=C_{p-raw,x}(t)+C_{p-raw,x}(t)\frac{\alpha }{2}\;\sin(2\pi \omega t).$$ (6)

To simulate the changes in the overall attenuation of arterial blood during vessel dilation and contraction, the absorption and scattering coefficients used in the time-resolved Monte-Carlo simulations were scaled to correspond to the relative change in arterial volume at each time step. This was done to avoid the high memory and computational requirements of simulating the true vessel diameter changes at each time step, due to the high resolution required to do so.

The simulated system, when employed clinically, would suffer from many imaging artifacts due to noise sources. To simulate this, the assumption was made that the combination of many noise sources could be approximated with simple Gaussian noise. Publicly available plethysmography signals taken from the fingers of patients at the Beth Israel Deaconess Medical Center (Boston, MA, United States),^43^ sourced via PhysioNet,^44^ were used to quantify the relative amount of Gaussian noise used here. To do so, a 1-min-long segment of the human signal was taken, and a 4th-degree polynomial fit was applied, where the standard deviation of the residuals between the true signal and the fitted signal was assumed to be representative of the variation seen in the human plethysmography data. This noise was then applied to this simulated signal by randomly sampling from the normal distribution, using MATLAB’s built-in randn() function, and scaled by the signal standard deviation found via the human data.

**Fig. 4:**
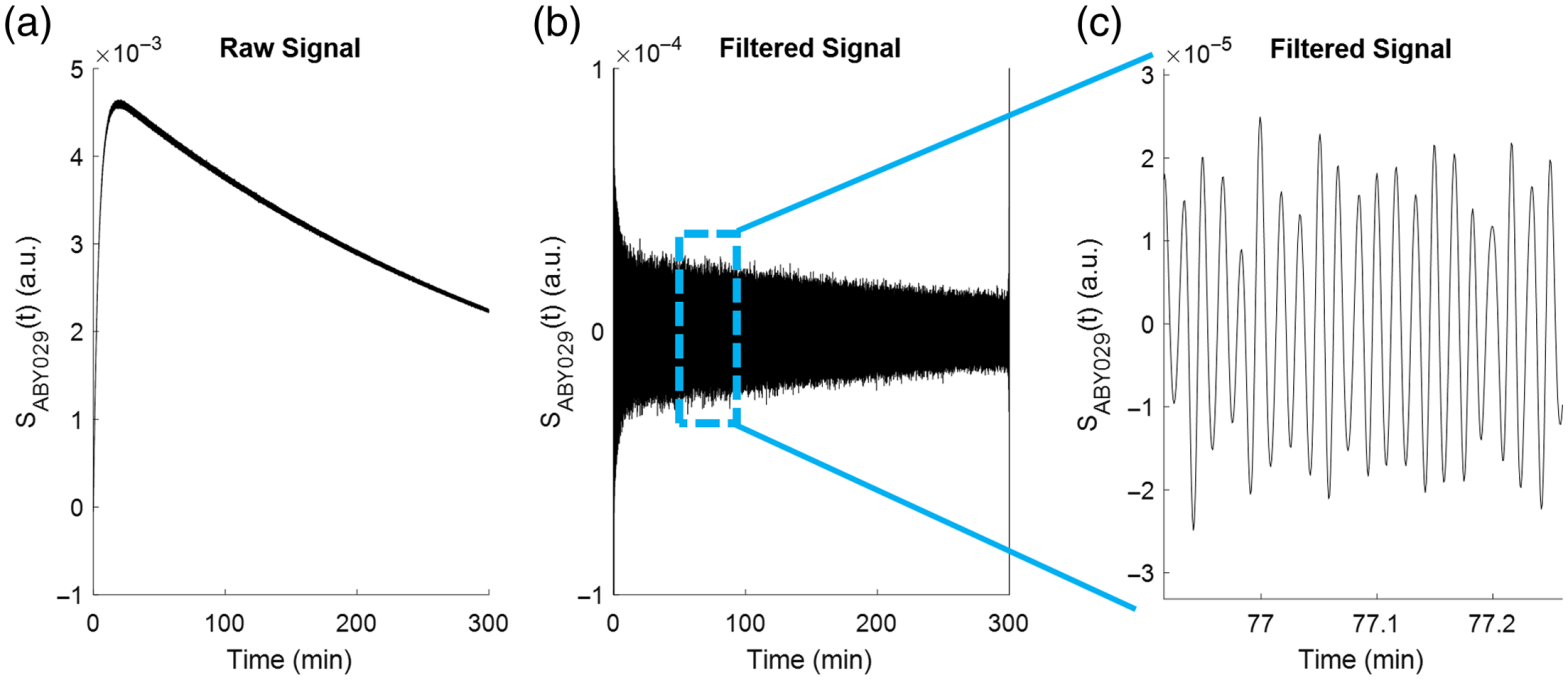
Example total signal (S(t)) curves for ABY-029 depicted in (a) its raw form, (b) after removal of the DC component, and (c) a magnified region of the signal, illustrating the signal pulses. The signal shown here was calculated directly from Eq. (5).

The total signal was simulated for 300 min with 50-ms step sizes [Fig. 4(a)], and a fast Fourier transform using the fft() algorithm in MATLAB was enacted on the simulated ABY-029 and IRDye 680LT signals to simulate a 10-Hz long pass filter and remove the signal’s DC-offset component [Fig. 4(b)]. A zoomed view of the pulsatile nature of the signal can be seen in Fig. 4(c). Finally, noise was added by scaling the maximum signal in each channel respectively to the half-maximum bit-depth of a 16-bit detector and adding randomly sampled noise from the Poisson distribution (assuming shot-noise) using the *poissrnd*() function in MATLAB.

### 2.2 Paired Agent Fluorescence Pulsed Dye Densitometry (PAF-PDD)

Over the course of the 300 min, the simulated signal was sampled every 50 ms across 1-min intervals, corresponding to a sampling rate of 20 Hz, commonly employed by clinical pulse oximeters.^45^ A Fourier transform was carried out on each 1-min interval using MATLAB’s fft() function. The frequency in the Fourier spectrum with the largest magnitude of its real component was then isolated for each of the intervals (corresponding to the heart rate) and saved in an array. This array was then normalized to its maximum value, resulting in the final reconstructed and normalized PIF as a function of time, with a resolution of 1 min. This resolution was chosen due to the assumption that the imaging agents have had enough time to mix in the blood (typically about 1 min) so that plasma concentration is similar among all blood regions.

### 2.3 Estimating the Validity of PAF-PDD in a Simulated Tumor Paired-Agent Binding Potential Study

#### 2.3.1 Simulation of targeted and untargeted imaging agent signals in a tumor

**Fig. 5:**
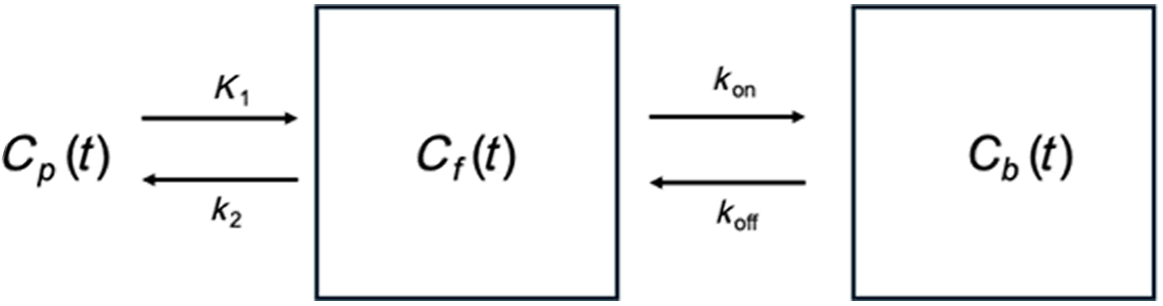
Two-compartment model used to simulate the imaging agent concentrations in a tumor-bearing individual, with the PIF per imaging agent, $C_{p}$, feeding the “free” imaging agent compartment ($C_{f}$), which in turn feeds into the “bound” imaging agent compartment ($C_{b}$). PIFs used can be seen in Eq. (7) for ABY-029 and Eq. (8) for IRDye 680LT. $K_{1}$ represents the extravasation of the corresponding imaging agent from the blood into the “free” compartment space, $k_{2}$ representing the flow back into the blood, $k_{\text{on}}$ describing ABY-29’s rate of binding to the receptor of interest, and $k_{\text{off}}$ representing its dissociation. For IRDye 680LT simulations, $k_{\text{on}}$ and $k_{\text{off}}$ were set to 0, effectively changing this two-compartment model into a single compartment. The corresponding differential equations describing the model are noted in Eqs. (5) and (6), with $B_{\max}$ representing the maximum concentration of receptors available for binding.

To assess the utility of this technique for use in PAI studies, “tumor” simulations were conducted using a two-compartment model to simulate the concentration profiles of ABY-029 and IRDye 680LT over time in both tumor “unbound,” $C_{f}(t)$, and “bound,” $C_{b}(t)$, compartments where the total fluorescence signal was calculated as the sum of the concentrations in the two compartments per time point. Tissue A depiction of the two-compartment model is presented in Fig. 5, and the corresponding system of ordinary differential equations can be represented as

$$\frac{dC_{f}}{dt}=K_{1}C_{p}(t)-k_{2}C_{f}(t)-k_{\text{on}}[B_{\max}-C_{b}(t)]C_{f}(t)+k_{\text{off}}C_{b}(t),$$ (7)

$$\frac{dC_{b}}{dt}=k_{\text{on}}[B_{\max}-C_{b}(t)]C_{f}(t)-k_{\text{off}}C_{b}(t),$$ (8)

where $k_{\text{on}}$ and $k_{\text{off}}$ represent rate constants governing the likelihood the ABY-029 binds to or dissociates from EGFR, respectively, and $B_{\max}$ represents the concentration of EGFR available for binding. $K_{1}$ and $k_{2}$ values used were those described in Sec. 2.1.1; $B_{\max}$ was set to 10 nM: that of the moderate EGFR-expressing human oral squamous cell carcinoma tumor line FaDu, calculated assuming an average cell volume of ∼5000 to $1000\;\mu m^{3}$ and 700 to 10,000 EGFR molecules per FaDu cell;^46^ and $k_{\mathrm{on}}$ and $k_{\text{off}}$ were set to previously described values, $0.05\;\min^{-1}.{\mathrm{nM}}^{-1}$ and $0.12\;\min^{-1}$, respectively.^2^ For the control imaging agent (IRDye 680LT) simulations, $k_{\text{on}}$ was set to zero. Cp(t) was created for ABY-029 and IRDye 680LT from Eqs. (1) and (2), respectively. MATLAB was used to solve the system of differential equations in Eqs. (7) and (8) using a fourth-order, five-step Runge-Kutta method through the built-in function *ode45*(). The resulting $C_{f}$ and $C_{b}$ concentrations simulated over 300 min at 1-min intervals were added together to simulate the total tissue concentration of both agents. The noise was added to the final curves using MATLAB’s built-in *poissrnd()* function, where it was assumed the noise was predominantly shot noise of a 16-bit detector.

#### 2.3.2 Deconvolution correction of the control imaging agent curve

Although the original paired-agent molecular imaging protocols developed by our group were built on the assumption that the PIFs of the targeted and control imaging agents were similar,^4^ a deconvolution approach was developed to correct for PIF differences.^17^ In brief, this method involved representing the difference in PIFs by expressing the targeted PIF ($C_{p,\text{targeted}}$) as equivalent to the control PIF ($C_{p,\text{control}}$), if the control PIF were convolved with an appropriate function g(t), as such

$$C_{p,\text{targeted}}(t)=C_{p,\text{control}}(t)*g(t).$$ (9)

Then, if g(t) could be estimated using Eq. (9) through “deconvolution,” the resulting estimate of g(t) could be convolved with all other measured concentrations of the control agent to correct for the PIF differences in all tissues of interest. In the past, the deconvolution step was always carried out at the level of a “reference” region—one devoid of the targeted biological molecule—owing to past challenges of directly measuring the PIFs. In simulations, g(t) was estimated from the noise-added ABY-029 and IRDye 680LT PIFs extracted from the simulated raw PAF-PDD data [Eq. (5)], as described in Sec. 2.2. The deconvolution operation was carried out in MATLAB using a singular value decomposition methods as described in detail previously.^17^

#### 2.3.3 Fitting of a simplified reference tissue model for BP

The previously published simplified reference tissue model (SRTM)^18^ [Eq. (10)] was fit to simulate ABY-029 and IRDye 680LT signals as a function of time as described in Sec. 2.3.1.

$$C_{\mathrm{tar}}(t)=R_{1}C_{\mathrm{con}}(t)*g(t)+[k_{2}-\frac{R_{1}k_{2}}{1+BP}]C_{\mathrm{con}}(t)*g(t)*e^{\frac{-k_{2}}{1+BP}t}.$$ (10)

The parameter $R_{1}$ represents the ratio of $K_{1}s$ for the targeted and control agents, respectively (in this work the value was 1), $C_{\text{tar}}(t)$ represents $C_{f}+C_{b}$ + noise from Sec. 2.3.1 using the ABY-029 simulation input parameters, and $C_{\mathrm{con}}(t)$ represents the same thing but using the IRDye 680LT simulation input parameters. Fits were carried out on four variations of simulated targeted and control imaging agent signals in the tumor, for a comparison of methods: (1) where the targeted and control agent signals were simulated with the same PIF [both having a PIF described by Eq. (1)]—the results from these were labeled “Equivalent PIFs”; (2) where imaging agent signals were simulated with differing PIFs and including the deconvolution correction [using Eqs. (1) and (2) for ABY-029 and IRDye 680LT PIFs]—the results from these were labeled “differing PIFs; perfect deconvolution”; (3) where the agent signals were simulated with differing PIFs [Eqs. (1) and (2) for ABY-029 and IRDye 680LT PIFs, respectively] and ignoring the deconvolution correction [removing g(t) from Eq. (10)]—the results from these were labeled “differing PIFs; no deconvolution”; and (4) where imaging agent signals were simulated with differing PIFs and including the deconvolution correction (estimating g(t) as described in Sec. 2.3.2)—the results from these were labeled “differing PIFs; PAF-PDD deconvolution.” Fitting was carried out in MATLAB using the nonlinear least-squares fitting function, *lsqcurvefit*().

### 2.4 Statistics

The accuracy and precision of the proposed PAF-PDD PIF estimation method (Sec. 2.2) for both imaging agents was evaluated by comparing the PAF-PDDs directly to the “true” PIFs [Eqs. (1) and (2)] using the mean-squared error (MSE) between curves across different dynamic range detector noise simulations. A paired-sample Student’s t-test was used to compare the resulting BP measurements between the cases where the simulated curves utilized differing PIFs, both with and without PAF-PDD correction (cases 3 and 4 described in Sec. 2.3.3, respectively), and the case where the curves were corrected via deconvolution with the tracers’ true PIFs (case 2 described in Sec. 2.3.3).

## 3 Results

### 3.1 Comparison of PAF-PDD Estimates of PIFs with the True PIFs

**Fig. 6:**
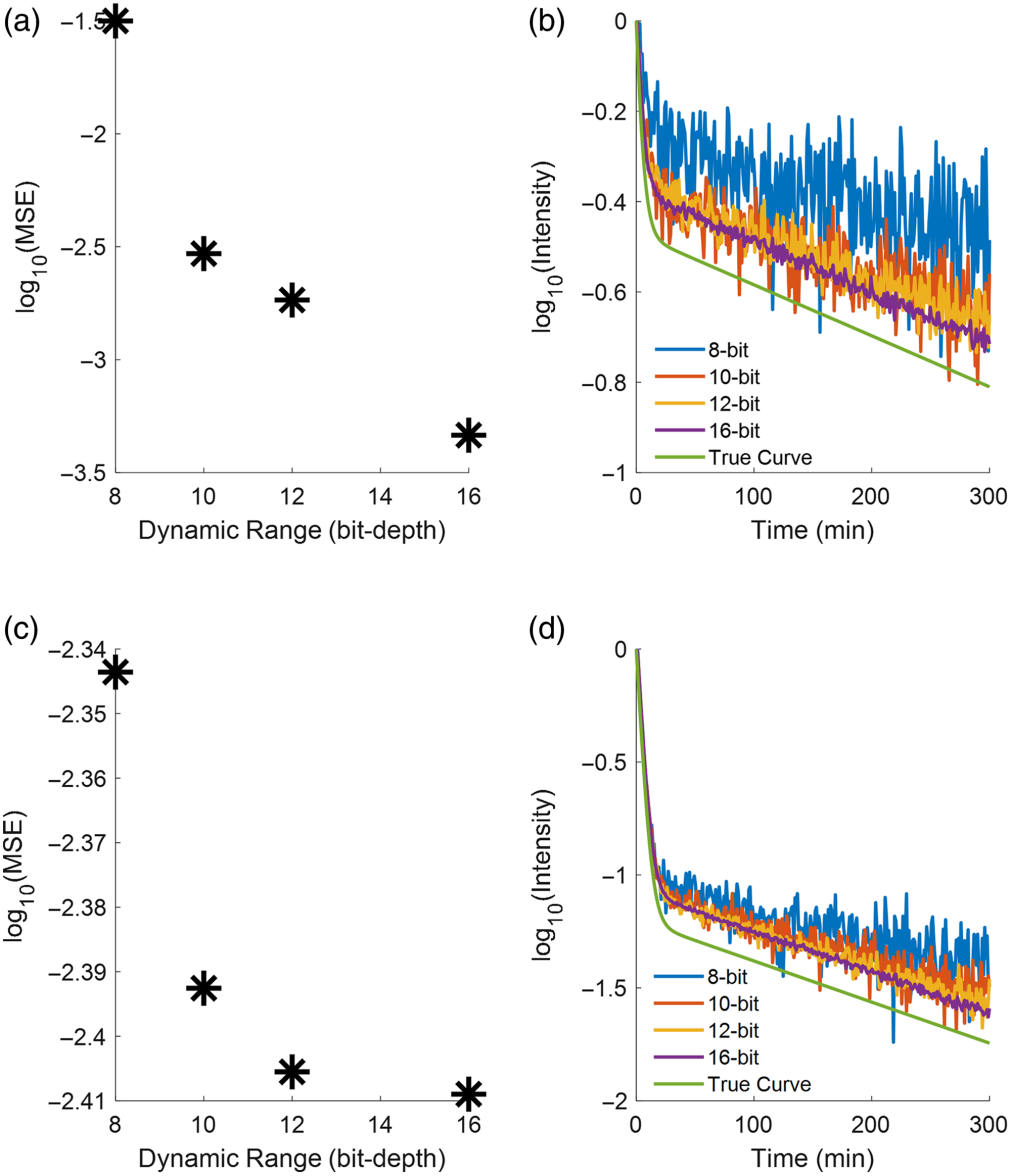
Resulting reconstructed relative PIFs (b, d, log-scale) at different bit-depth detector noise levels for ABY-029 (a, b) and IRDye 680LT (c, d), with the mean squared error between a reconstructed curve and true curve [as calculated from Eqs. (1) and (2)] as a function of bit-depth (a, c, log-scale).

Comparison between PAF-PDD estimates of the PIFs for both imaging agents ABY-029 and IRDye 680LT and the “true” PIFs, which were developed from Eqs. (1) and (2) and were used as input in 4 to 6 to simulate the PAF-PDD data, was carried out at a range of noise levels (assuming 8-bit to 16-bit detector dynamic range, full use of the dynamic range, and that noise was dominated by shot noise/Poisson noise). The solutions to Eq. (1) (typical PIF of ABY-029 in mice) and 2 (typical PIF of IRDye 680LT in mice) are plotted in Figs. 6(b) and 6(d), respectively, along with 8-, 10-, 12-, and 16-bit detector dynamic range PAF-PDD estimates of the corresponding PIFs. Across the common dynamic ranges (as in those dynamic ranges readily seen in commercially available scientific cameras) evaluated, a simulated 8-bit detector yielded reconstruction of the relative PIFs with a calculated MSE of $3\times 10^{-2}$ and $5\times 10^{-3}$ for ABY-029 and IRDye 680LT, respectively, whereas the 10-bit-detector simulations yielded MSEs of $3\times 10^{-3}$ and $4\times 10^{-3}$; the 12-bit-detector simulations yielded MSEs of $2\times 10^{-3}$ and $4\times 10^{-3}$; and the 16-bit-detector simulations yielded the lowest MSEs of $6\times 10^{-4}$ and $4\times 10^{-3}$ for the ABY-029 and IRDye 680LT PIF shape reconstruction accuracies, respectively.

### 3.2 Comparison between Fit BP Values

**Fig. 7:**
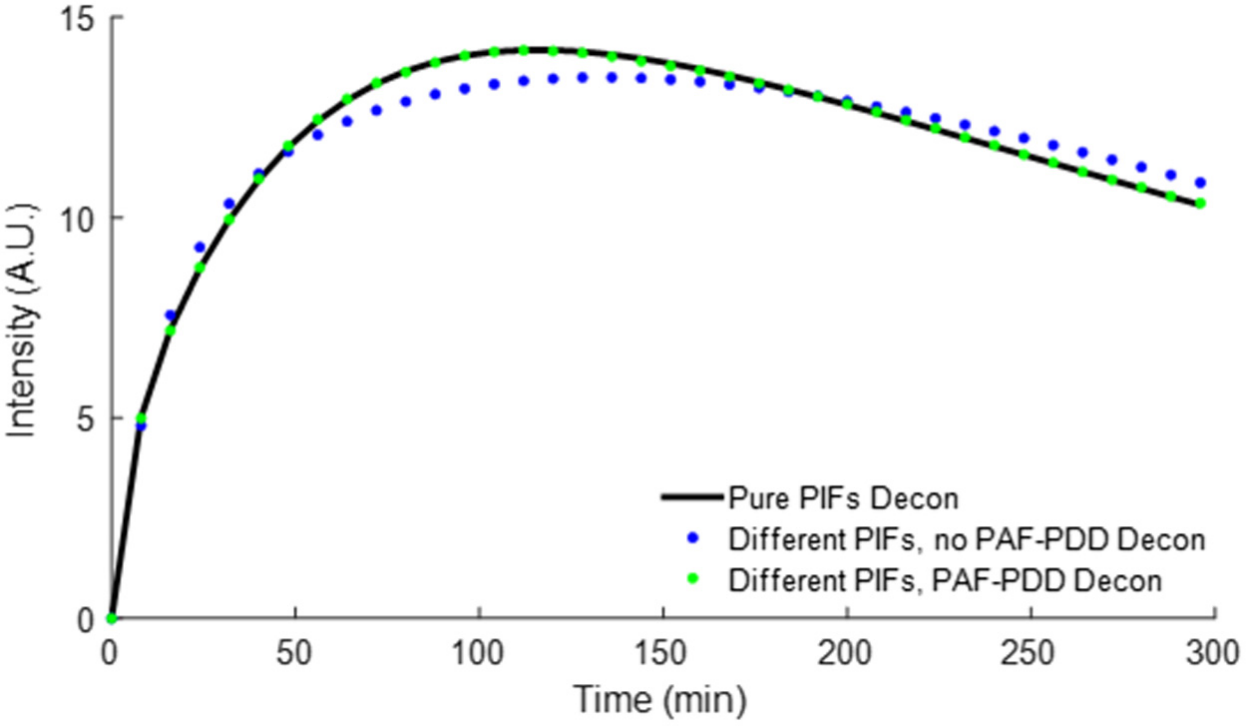
Results of fitting the SRTM using targeted agent curves deconvolved from the pure analytical PIFs (black), using no deconvolution correction (blue), and deconvolution using the measured PIFs via the proposed PAF-PDD method (green).

The MSE measurements in Sec. 3.1 provided a means of assessing the relative improvement of PIF estimation using detectors with higher resolution and ensuring the PAF-PDD system is designed to make use of the full bit-depth. However, these results on their own provided little insight into what level of MSE is necessary to adequately use PAF-PDD for PIF estimation with the purpose of correcting for targeted and control agent PIF differences in paired-agent SRTM experiments aimed at quantifying targeted biomolecule concentration in tissue via the BP parameter. The noise-added simulated targeted and control agent dynamics in a tumor, as described in Sec. 2.2, are shown in Fig. 7. Fit results for four cases detailed in Sec. 2.3.3, with the corresponding extracted BP values and their relative error from simulated curves using the same PIF as “truth,” are presented in Table 2. As expected, the use of no convolution correction in the final BP fit resulted in a final error of 691.0±0.8% (24.08±0.02 compared with 3.04 as “truth”). This is in comparison to using the reconstructed PIF curves as an input to the convolution correction algorithm, resulting in a final BP error of only 2±1% (3.12±0.04 compared with 3.04 as “truth”).

**Table 2:** Resulting BP values from fitting imaging agent curves to the SRTM with simulated curves where agents possess the same PIF (column 1), a deconvolution correction using the true PIFs for the imaging agents (column 2), no deconvolution correction (column 3), and the correction using the reconstructed curves from the methodology presented here (PAF-PDD, column 4).

	Equivalent PIFs	Differing PIFs; perfect deconvolution	Differing PIFs; no deconvolution	Differing PIFs; PAF-PDD deconvolution
**BP**	3.040 ± 0.002	3.09 ± 0.02	24.08 ± 0.02	3.12 ± 0.04
**Relative error**	0.00043 ± 0.0003	0.014 ± 0.006	6.91 ± 0.01	0.024 ± 0.01

Relative errors shown represent the deviation of the fit BP value per method with the deconvolution correction using the true PIFs as the “true” BP value, where in this case, no noise was added to the curves. All simulations presented here were conducted across 10 noise iterations using a Poisson noise model mimicking a 16-bit detector. In the case of equivalent and different PIFs (no deconvolution correction), the mean and standard deviation represent variation due to noise addition, whereas in the case of different PIFs using the proposed PAF-PDD, variation presented is due to noise addition in both the simulated tumor curves as well as simulated and reconstructed PIFs via the PAF-PDD methodology.

A Student’s t-test was used to determine whether a statistical difference existed between the average BP estimated using the analytical expressions for the simulated PIFs for ABY-029 during deconvolution correction (method 2 described in Sec. 2.3.3) and the BPs estimated via both no deconvolution correction (method 3 described in Sec. 2.3.3) as well as using the reconstructed PIFs via the proposed PAF-PDD methodology (method 4 described in Sec. 2.3.3). A statistical difference was found between the correction using the true PIFs and no deconvolution correction ($p<1\times 10^{-28}$), with the 95% confidence interval of their difference being calculated as [20.98, 21.01], whereas no statistical difference was found between BP estimated via deconvolution with the true PIFs and the BP estimated via deconvolution of curves with the proposed PAF-PDD methodology (p=0.14), with the 95% confidence interval of the difference between the two BP estimates calculated to be [−0.01,0.05].

## 4 Discussion

The ability to accurately quantify BP in paired-agent molecular imaging studies is dependent on correcting for differences in the PIFs of the targeted and control imaging agents. This work demonstrated, through simulations of photon propagation and imaging agent pharmacokinetics, that a modified pulse dye densitometry approach utilizing two fluorescent imaging agents (PAF-PDD) is feasible for reconstructing the relative PIF shapes of a targeted and control imaging agent administered concurrently to the level of accuracy that BP can be estimated within 5% of the truth. Specifically, the PAF-PDD–derived PIFs enabled accurate estimation of BP, with only a 1% error compared with BP estimated when using the true PIFs for simulation. By contrast, failing to account for PIF differences between the agents resulted in a 691% error in BP. These results highlight the importance of correcting for PIF differences in PAI and provide strong evidence that PAF-PDD is a promising approach for enabling such correction.

Traditional methods of capturing imaging agent PIFs involve blood sampling from populations of individuals, which is both invasive and challenging to collect frequently enough for accurate deconvolution correction of agent signals during kinetic analysis of time series data.^47^^,^^48^ To circumvent this, other groups have explored the use of alternative methods of recovering imaging agents’ PIFs on an individual basis. In the past, our group described a method of capturing fluorescent agent PIFs via direct exposure and imaging of a carotid artery, from which either fluorescence curves can be normalized and used directly in kinetic curve correction, or alternatively via the use of a calibrating standard curve to directly calculate the true concentration in the blood as a function of time.^21^ However, given the highly invasive nature of this technique, it is not amenable to use in humans and thus is unable to help with the transition of quantitative PAI modalities to the clinic. Other methods that have been proposed combine anatomical and molecular imaging methods so that anatomical details can be used prior to extracting blood signals from vessels and heart chambers in tomographic molecular imaging.^49^^,^^50^ More recently, a fluorescence PDD methodology was developed for the assessment of individual ICG PIFs for use in parameter estimation during perfusion studies.^51^^,^^52^ Although this method also employed low-cost and readily available equipment, its use was limited to only the first few minutes of post-injection as the method assumed all of the signal was only in the blood. This is not as much of a concern when using a highly protein-binding tracer such as ICG, where its retention in the plasma lowers its overall extravasation into the tissue;^53^ however, this is less appropriate for imaging agents that extravasate into the tissue. Furthermore, kinetic parameter estimation in PAI studies generally requires PIF measurement over the course of several hours, limiting this methodology’s applicability to PAI kinetic analysis.

The PAF-PDD method described in this work has the advantage that it can be used over many hours to track PIF signals, it does not require any blood sampling, and it can feasibly be implemented clinically by adapting existing pulse oximetry devices designed for fingers. Although PAI has not yet been translated to clinical studies,^3^^,^^20^ preclinical work has demonstrated that it can provide significant improvements in molecular sensitivity^9^ and tissue discrimination,^54^ particularly for cases of fluorescence-guided surgery, compared with single-agent imaging approaches, and there are several ongoing efforts to translate PAI to the clinic.

The simulations in this study were optimized based on the anatomy and physiology of human fingers, assuming physiologically relevant optical properties and using validated open-source photon propagation modeling toolkits.^32^^,^^55^^,^^56^ Experimentally relevant levels of noise were added; however, heart rate was not simulated to change during simulated experiments, only because it was challenging to accurately model a changing heart rate. Changing heart rate was simulated in a piece-wise way (results not shown), and it was found that if the heart rate was relatively stable over each 1-min data collection period, the results of this study would be unaltered. In the eventual development of a PAF-PDD device, a heart rate monitor could be installed with the device such that data collected in periods of rapid heart rate change could be extracted from data analyses.

For this initial feasibility test, we assume sinusoidal, periodic oscillations in Cp; however, in practice, the frequency and phase of the heart rate can change during measurements. In addition to this, signals extracted from the finger may suffer from motion artifacts. A key advantage of the method described here, however, is the use of a Fourier transform over 1-min-long segments of the signal. Because of this, changes that occur more rapidly than this stretch of time should be accounted for in the final reconstructed PIF. Future work will explore correction methods for changes that are more dynamic, and solutions in pulse-oximetry can be leveraged, as the artifacts are likely to be similar.

Another potential limitation of the Monte Carlo simulations employed in this work is that the modeling of the arterial dilation and contraction was simplified. Rather than dynamically changing the diameter of the vessel in the model, an approximation was used where the optical properties were adjusted in proportion to the change in blood volume occurring with each pulse. This approximation was necessitated by the high computational demands of modeling both the spatial and temporal changes associated with the cardiac cycle at sufficiently high resolution. However, given that any changes in photon fluence during arterial volume changes are attributable to changes in the number of scattering and absorption events, which can also be represented by increasing or decreasing the tissue optical properties proportionally, this is expected to be a reasonable approximation for the purposes of demonstrating the feasibility of PAF-PDD. Despite the focus on clinical translation in this work, the PAF-PDD approach could potentially be extended to preclinical imaging studies in rodents as well. Mice and rats have readily accessible ventral tail arteries that could be amenable to PAF-PDD measurements because more conventional PDD methods have been adapted to mouse imaging.^57^ Imaging the vasculature of the mouse ear is another possibility. However, the much faster heart rates of mice and rats would necessitate rapid data acquisition, requiring lower exposure times that could limit sensitivity. Further simulations and empirical studies would be needed to optimize the PAF-PDD methodology, including hardware specifications, for rodent imaging.

This work investigates the deployment of higher bit-depth detectors and the subsequent effects on the reconstruction of the PIF. It is important to clarify that the noise relating to a sensor’s bit-depth is largely dominated by quantization noise and shot noise. In the case of a shot-noise limited detection system, shot-noise can be approximated by randomly sampling the Poisson distribution. This type of noise is due to the quantum mechanical nature of electron transport across the sensor circuitry. This, however, is distinct from quantization noise, which is what is largely captured here. This kind of noise is due to the quantization of a continuous signal, requiring rounding of the signal to the nearest integer to yield a digital representation. To this end, the effects of this quantization noise become more evident when utilizing a lower bit-depth sensor and become less apparent as the bit-depth increases, as demonstrated in the noise analysis presented here.

In addition, the PIFs presented here do not have a “0” concentration at time 0, as would be seen clinically. This is because standard measurements of PIFs typically result in the fitting of a bi-exponential model of plasma kinetics [as seen in Eqs. (1) and (2)]. Using this fit model results in a calculated signal that does not begin at 0 but instead at the curve’s maximum. However, for the purpose of this proof of concept, it is not expected that the inclusion of this time 0 point in the PIF to be required for proper deployment of the technology detailed in this work. Further, it is important to note that PAF-PDD as presented here only provides relative PIF shapes, not absolute concentrations. Obtaining true concentration curves would require precise knowledge of the arterial volume changes during data acquisition, which is likely to vary between individuals. One potential solution could be to incorporate an additional LED emitting at a longer red or NIR wavelength where there is minimal absorption by the fluorescent imaging agents. In principle, changes in blood vessel diameter could be estimated from changes in hemoglobin absorption measured at this additional wavelength using the Beer-Lambert law. However, this approach was beyond the scope of the current work.

## 5 Conclusion

This study demonstrates the feasibility of utilizing PAF-PDD to reconstruct the relative PIFs of targeted and control imaging agents for quantitative PAI. Simulations show that the PAF-PDD-derived input functions can enable accurate estimation of BP, a key measure of molecular target density in tissues. By providing a noninvasive approach for PIF measurement in humans, PAF-PDD has the potential to greatly expand the applications of PAI in clinical research and molecular diagnostics. Further studies are warranted to translate this promising technique to in vivo imaging.

## Data Availability

All code, data, and materials used in this project and analysis can be obtained by direct email to the corresponding author, Dr. Kenneth M. Tichauer at ktichaue@iit.edu.
